# Bleomycin-Induced Lung Injury After Intravenous Iron Administration

**DOI:** 10.7759/cureus.27531

**Published:** 2022-07-31

**Authors:** Nicole Saccone, Jessica Bass, Michele L Ramirez

**Affiliations:** 1 Internal Medicine, NCH (North Collier Hospital) Healthcare System, Naples, USA; 2 Hematology and Oncology, Florida Cancer Specialists, Naples, USA

**Keywords:** iron chelation, intravenous iron supplement, iron deficiency anemia (ida), bleomycin-induced lung injury, bleomycin-induced pneumonitis

## Abstract

Bleomycin is an antibiotic that is often used as a chemotherapeutic agent due to its antitumor activities against a variety of malignancies. A feared and often fatal side effect of this drug is a pulmonary injury causing inflammation that can progress to pulmonary fibrosis. Bleomycin damages lung endothelial cells by the production of free radicals that can only occur when it is bound to certain metals in the body. It forms a complex with iron and once activated by iron reduction, it destroys deoxyribonucleic acid (DNA). Therefore, it is suggested that the availability of iron in the body may play a role in the pathogenesis of bleomycin toxicity although no related cases have been documented. This is a case of a 75-year-old female with no prior history of pulmonary disease who was diagnosed with Hodgkin’s lymphoma and received 12 doses of bleomycin over the course of six cycles of chemotherapy. She then presented to the hospital with respiratory failure five months after her completion of treatment. Interestingly, one month prior to presentation, she was given two intravenous iron infusions of ferumoxytol five days apart for the treatment of iron deficiency anemia. Within a week of receiving the iron, she developed dyspnea with a nonproductive cough. About a month after she developed these symptoms, she presented to the hospital and was found to be hypoxic with any activity and was subsequently placed on oxygen via nasal cannula. Her lung imaging showed new reticulonodular and patchy infiltrates bilaterally concerning for pneumonitis and her physical examination was significant for black discoloration of her fingertips and toes along with expiratory rhonchi heard throughout her lungs. During the hospitalization, her oxygen requirements increased, and the patient ended up in the intensive care unit on bilevel positive airway pressure. Her lung imaging, rapid progression, and skin findings made the clinical diagnosis of bleomycin toxicity. Out of concern that the intravenous iron may have played a role in the toxicity, iron chelation was attempted. The patient was given two doses of deferoxamine over two consecutive days and her symptoms of dyspnea along with her oxygen requirements improved. Unfortunately, these positive effects only lasted a few days and the patient continued to decline and ultimately passed away.

This case raises many questions regarding iron’s role in bleomycin toxicity, including if intravenous iron infusions may increase the risk of pulmonary injury from bleomycin. There are currently no guidelines or recommendations that suggest withholding iron supplementation in patients undergoing chemotherapy with bleomycin. There is also insufficient evidence to support the routine use of iron chelation in a patient that presents with bleomycin-induced lung injury. However, some studies suggest that iron chelation may play a role in preventing pulmonary toxicity. It may also lessen the severity of the toxicity or improve some of the related symptoms, thus warranting further research.

## Introduction

The bleomycin family of antibiotics was first isolated in 1966 and is produced by the organism Streptomyces verticillus. It is commonly used to treat lymphomas and germ cell tumors but can also be used to treat Kaposi’s sarcoma and some squamous cell carcinomas. Several pulmonary syndromes are associated with its use and dermal hyperpigmentation, and/or skin fibrosis can also occur [1].

Pulmonary toxicity is the most feared complication of bleomycin therapy and is relatively common. Approximately 10-35% of patients receiving adriamycin, bleomycin sulfate, vinblastine sulfate, and dacarbazine (ABVD) therapy for Hodgkin’s lymphoma develop some pulmonary toxicity, and 4-5% progress to fatal fibrosis [2]. Importantly, some patients with bleomycin-induced pneumonitis may recover and have reversible lung damage without progression to fibrosis [1]. Some risk factors for bleomycin toxicity include age older than 70, impairment of renal function, smoking, or increased oxygen exposure most likely from undergoing a procedure [3]. The toxicity may occur up to six months after treatment with bleomycin, and given that we are limited in reducing these known risks to prevent fatal fibrosis, we need to consider the clinical relevance of iron [1].

Bleomycin binds to multiple metals including iron, copper, cobalt, and others [4]. The bleomycin molecule has two structural components that integrate into host deoxyribonucleic acid (DNA), one of which binds iron and oxygen. Once bound, this complex (O2-Fe (II)-bleomycin) becomes activated and can release damaging oxidants, causing the destruction of the DNA in lung endothelial cells [5]. In this way, iron may be a key player in the ability of bleomycin to act as a toxic agent and may be an integral component of the physiologic cascade leading to pulmonary injury. This possibly argues against iron supplementation for patients receiving bleomycin or making the case of iron chelation in bleomycin toxicity. However, the clinical significance currently remains unknown. Data on humans are severely lacking and upon a thorough literature review, no case reports on this topic were found.

We, therefore, present a case of bleomycin-induced lung injury occurring four months after the discontinuation of bleomycin with sudden onset after the administration of intravenous iron and with symptom improvement after iron chelation therapy.

## Case presentation

A 75-year-old female with a history of idiopathic thrombocytopenic purpura, coronary artery disease, hypertension, dyslipidemia, type 2 diabetes mellitus, sarcoidosis, and Hodgkin's lymphoma diagnosed 10 months prior presented with complaints of a dry cough that had been present for one month with progressive dyspnea on exertion that had worsened in intensity. She noticed that morning while she was shopping that she had difficulty catching her breath with minimal activity. She denied recent illnesses or sick contacts and was up to date on her vaccinations. She also denied chest pain, palpitations, fever, chills, nausea, vomiting, diaphoresis, sore throat, dysuria, rashes, numbness or tingling in upper or lower extremities, weakness, headache, dizziness, or visual changes.

**Figure 1:**
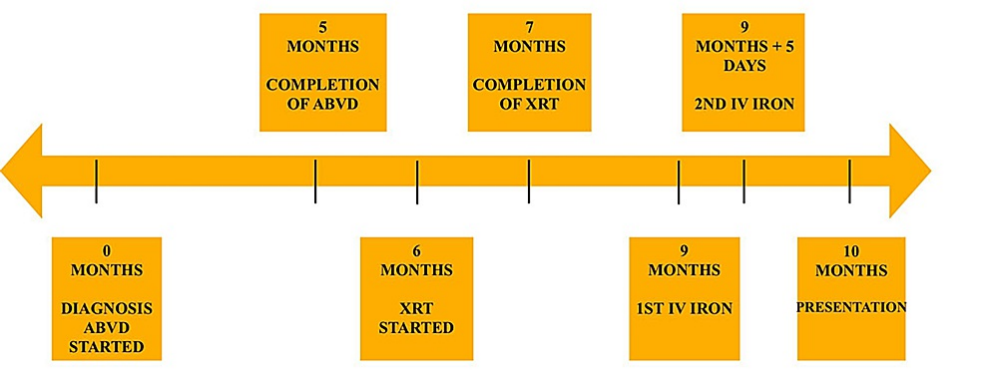
Timeline displaying time of her Hodgkin’s lymphoma diagnosis, when she began and ended ABVD therapy, when she began and ended XRT, when she received IV iron and when she presented to the hospital with respiratory symptoms. ABVD, adriamycin, bleomycin sulfate, vinblastine sulfate, dacarbazine; XRT, radiation therapy; IV, intravenous.

The patient’s Hodgkin’s lymphoma was previously treated with six cycles of ABVD therapy over the course of five months and then 18 doses of right axillary radiotherapy (XRT). She received 16 units of bleomycin sulfate with each ABVD cycle (12 total doses) and tolerated the regimen well. A positron emission tomography (PET) scan during cycle 5 showed stable disease with no new evidence for active lymphoma. Four months later, she reported to her oncology team that she was having severe restless legs and was worked up for anemia. She was diagnosed with iron deficiency anemia and was given two doses of 510 mg ferumoxytol intravenously separated by five days in the outpatient setting (Figure 1).

A PET scan performed four days prior to presentation at an outside facility showed stable lymphadenopathy and new diffuse bilateral pulmonary infiltrates. She did not have a previous history of pulmonary disease or occupational exposure but was a former smoker of 30 pack-years who quit 20 years prior to presentation.

Vitals upon arrival to the emergency room were significant for a heart rate of 105 beats per minute and oxygen saturation of 92% on room air. The patient was placed on 3 liters via nasal cannula and her saturation improved to 100%, though she was noted to still be tachypneic and dyspneic with speech and desaturated with minimal activity. Physical examination was significant for expiratory rhonchi bilaterally heard both anteriorly and posteriorly. Her fingers and toes were black at the tips which was a new finding. Labs were significant for a hemoglobin of 11.9 g/dL, chronic platelet count of 38 × 10^3^/uL, D-dimer 1.18 ug/mL FEU, and negative troponin (Table 1).

**Table 1:** Lab values present on admission with lab tested in column 1, lab value of the patient in column 2, and normal lab values at our institution in column 3. WBC, white blood cells; RBC, red blood cells; HGB, hemoglobin; HCT, hematocrit; MCV, mean corpuscular volume; BUN, blood urea nitrogen; LDH, lactate dehydrogenase; ALK Phos, alkaline phosphatase; ALT, alanine transaminase; AST, aspartate transaminase; ACE, angiotensin-converting enzyme.

Lab Tested	Patient's Lab Values	Normal Values
Troponin	<0.05 ng/mL	≤0.15 ng/mL
WBC	6.5 × 10^3^/uL	4.2-10.8 × 10^3^/uL
RBC	4.19 × 10^6^/uL	3.70-4.90 × 10^6^/uL
HGB	11.9 g/dL	12.0-16.0 g/dL
HCT	36.3%	37-47%
MCV	87 fL	80-100 fL
Platelets	38 × 10^3^/uL	130-450 × 10^3^/uL
Na	139 mmol/L	136-145 mmol/L
K	3.5 mmol/L	3.5-5.1 mmol/L
Cl	106 mmol/L	98-107 mmol/L
CO_2_	28 mmol/L	21-32 mmol/L
Glucose	117 mg/dL	70-99 mg/dL
BUN	8 mg/dL	7-18 mg/dL
Cr	0.6 mg/dL	0.6-1.3 mg/dL
LDH	244 IU/L	84-246 IU/L
Albumin	2.3 g/dL	3.4-5.0 g/dL
ALK Phos	168 IU/L	50-136 IU/L
ALT	11 IU/L	12-78 IU/L
AST	15 IU/L	15-37 IU/L
Ca	9.0 mg/dL	8.5-10.1 mg/dL
ACE	34 U/L	9-67 U/L
Procalcitonin	<0.05 ng/mL	≤0.05 ng/mL
D-Dimer	1.18 ug/mL FEU	0.00-0.60 ug/mL FEU

**Figure 2:**
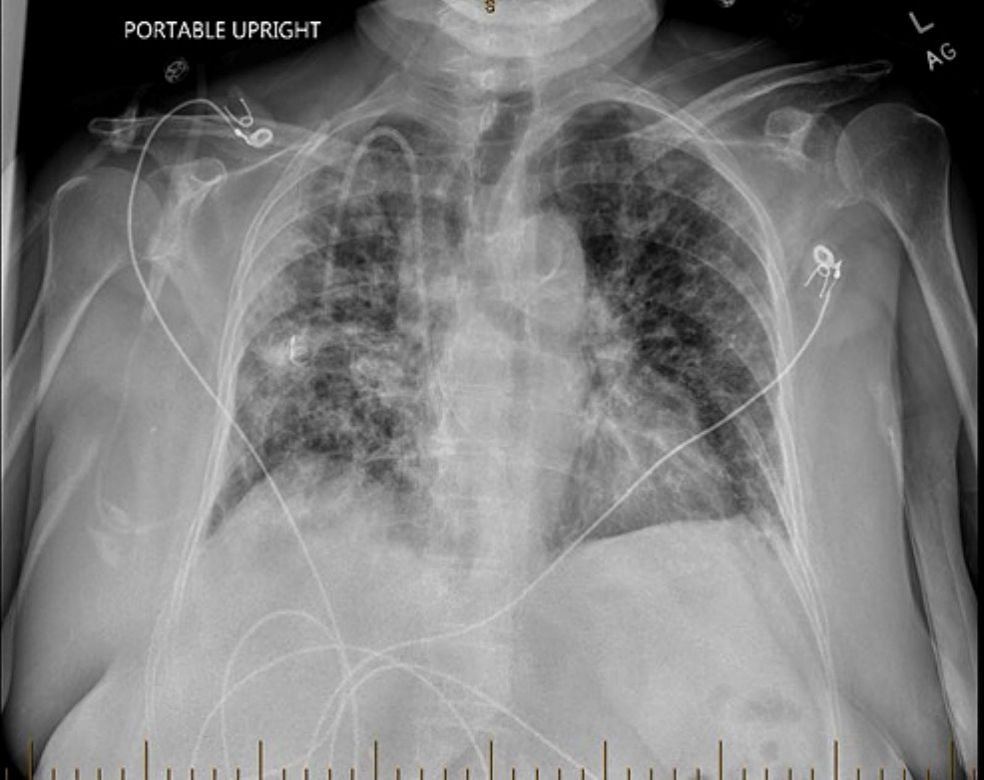
Portable chest X-ray on the day of admission showing new patchy infiltrates bilaterally.

**Figure 3:**
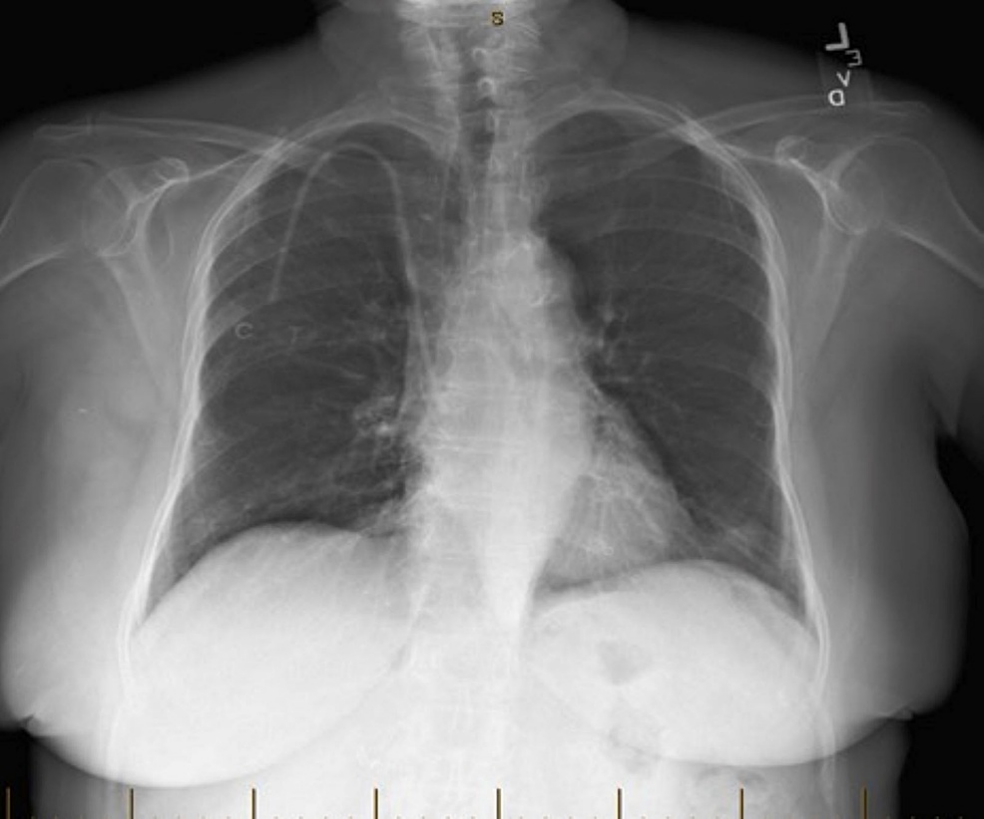
Portable chest X-ray taken 10 months prior to the day of admission that shows absence of the patchy infiltrates seen in Figure 2.

**Figure 4:**
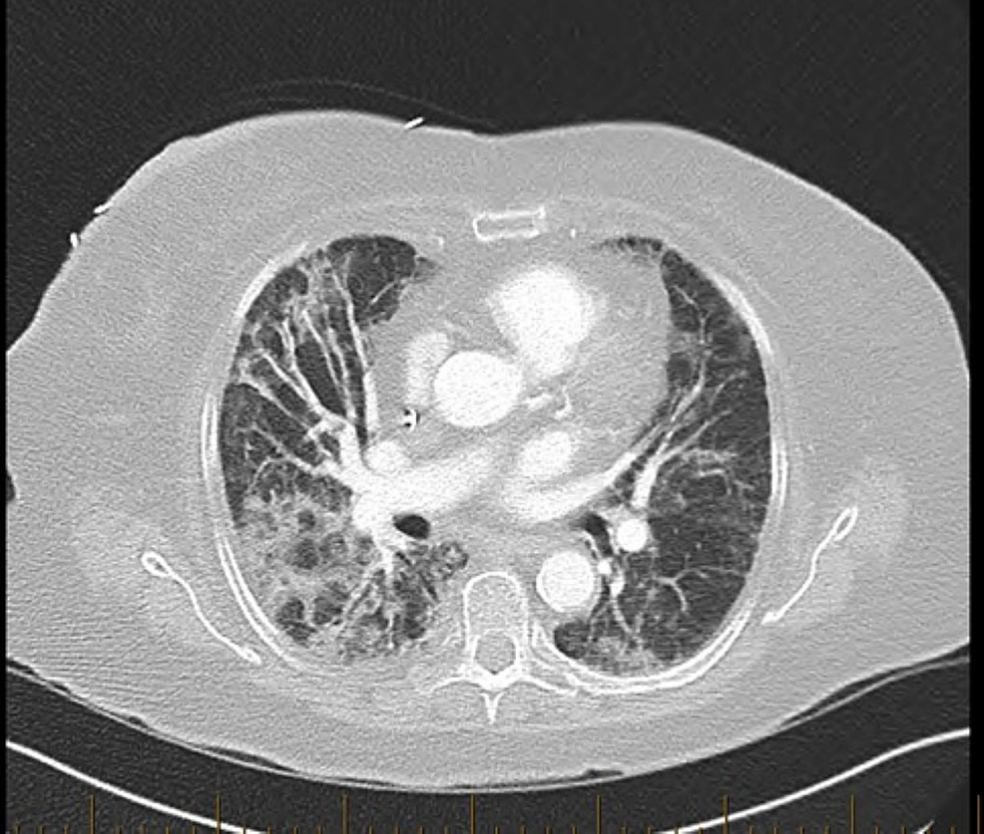
Computed tomography angiography (CTA) of the chest taken on the day of admission showing bilateral diffuse parenchymal opacities right greater than left with interlobular septal thickening and some mild central bronchiectasis.

Blood cultures were collected and were negative. The full PCR respiratory panel that included COVID-19 was negative (Table 2). EKG on arrival showed new first-degree atrioventricular block but no signs of right or left heart strain, and an echocardiogram was not remarkable for significant pathology as it showed a preserved ejection fraction and normal pulmonary artery systolic pressure. A portable chest X-ray on the day of admission showed reticulonodular and patchy infiltrates throughout both lung fields suspicious of widespread pneumonitis that was not present on previous imaging 10 months prior (Figures 2, 3). Computed tomography angiography performed on the same day showed bilateral diffuse parenchymal opacities right greater than left with interlobular septal thickening and some mild central bronchiectasis (Figure 4).

**Table 2:** Viruses that were tested for and found to be negative on our in-house respiratory viral panel.

Virus Tested	Result
SARS-CoV2 virus	Negative
Adenovirus DNA	Negative
Bordetella parapertussis	Negative
Bordetella pertussis ptxP	Negative
Chlamydia pneumoniae	Negative
Influenza A	Negative
Influenza B	Negative
Human metapneumovirus	Negative
Mycoplasma pneumoniae	Negative
Parainfluenza 1-4	Negative
Respiratory syncytial virus	Negative
Rhino enterovirus	Negative
Coronavirus HKU1	Negative
Coronavirus NL63	Negative
Coronavirus OC43	Negative
Coronavirus 229E	Negative

Pulmonology and oncology were consulted. Differential diagnoses included infection versus drug-induced pneumonitis versus radiation-induced pneumonitis versus reactivation of sarcoidosis. Antibiotics were empirically started in the emergency room but were discontinued on hospital day 3 as the infection was unlikely given the absence of fever, leukocytosis, or elevated procalcitonin. Calcium and angiotensin-converting enzyme levels were ordered and were within normal limits, making her history of sarcoidosis unlikely to be contributing to the current presentation (Table 1).

Prednisone 60 mg daily was initiated with the intention of treating suspected pneumonitis. The patient remained on 3 liters of oxygen via nasal cannula for a few days, but she was still dyspneic and unable to speak more than 2-3 words at a time despite the steroids. Pulmonology then placed her on heated high flow and adjusted her positioning in the bed by having her lay on each side to see if it would help her respiratory status. However, her breathing continued to decline.

Oncology assessed the patient and highly suspected bleomycin-induced lung toxicity given her clinical presentation of worsening dyspnea on exertion, dermal hyperpigmentation seen on all digits, pulmonary imaging, and timeframe with regard to bleomycin administration. They recommended a goal of oxygen saturation between 89% and 90% to not worsen the bleomycin toxicity and therefore decreased her supplemental oxygen. The patient continued to desaturate into the mid-80s at rest and the 50s with any activity and the decision was made to move her to the ICU for bilevel positive airway pressure (BiPAP) therapy and closer monitoring. Steroids were continued. Since the patient had recently received iron infusions and a possible mechanism of bleomycin toxicity involves the role of iron in producing free radicals causing oxidative damage, the decision was made by oncology to attempt iron chelation. The patient received 500 mg of intravenous deferoxamine infused over 8 hours on day 5 of admission and on day 6 she was re-evaluated. Her subjective dyspnea had improved but she remained on BiPAP. Given her improvement in comfort, the decision was made to give an additional dose of 500 mg of deferoxamine that day. The following morning, the patient’s oxygen saturation improved, and she was taken off BiPAP and switched to heated high flow on 35 liters with a fraction of inspired oxygen of 40%. She reported that she was feeling significantly better and was able to speak in full sentences without difficulty. She remained stable and more comfortable for a couple of days. Unfortunately, her oxygen requirements increased again, and the patient and her family decided to pursue inpatient hospice where she ended up passing a few days later, 15 days after admission.

## Discussion

Our patient presented with classic findings of bleomycin-induced pneumonitis with a nonproductive cough, tachypnea, and exertional dyspnea that progressed to dyspnea at rest [1]. She also had evidence of both lung and skin involvement with the hyperpigmentation seen on her fingers and toes appearing just prior to presentation. An interesting point in this patient is the timeline of events. She had completed ABVD therapy four months prior to presentation and XRT three months prior to presentation and tolerated each well. However, within one month of presentation and within days of IV iron administration, she began to have symptoms of shortness of breath and dyspnea followed by dermal hyperpigmentation. PET performed days prior to admission was starkly contrasted with her PET performed after completion of ABVD therapy months ago. This sharp change in the course despite no additional bleomycin being given suggests an inciting event and the timing of iron administration makes it likely that it was related to the toxicity in some capacity. Furthermore, both her subjective and objective improvement after iron chelation therapy suggests that iron may play a clinically significant role.

The mechanism of lung injury in bleomycin toxicity is believed to be from the free radical formation which involves iron as an activating component [5]. It has been suggested that bleomycin acts as a ferrous oxidase which promotes the toxicity of iron and that iron by itself can be toxic and can degrade DNA [4]. Therefore, bleomycin directly interacts with iron to cause cell toxicity and the availability of iron in the body may play a large role in the pathogenesis of bleomycin-induced lung injury; however, clinical studies in human subjects are severely lacking. A study was found in hamsters that looked at the effect of iron deficiency on bleomycin-induced lung injury. They induced mild iron deficiency by bleeding the hamsters and maintaining them on an iron-depleted diet. The iron-deficient hamsters then received bleomycin and they did not see the accumulation of lung collagen occur when compared to iron-replete hamsters that had significant increases in their lung collagen. Their study data suggested that iron deficiency is associated with a reduction in the severity of bleomycin-induced lung fibrosis [6]. Our patient had iron deficiency anemia when she was being treated with bleomycin. The findings above propose that this may have been a protective measure that would have made the lung toxicity less likely in her case had she not received the iron repletion. Knowing the answer to this may have changed the course of her disease and may prevent others from suffering these complications in the future.

A separate hamster model study attempted injecting deferoxamine into hamsters that were given bleomycin to see if it reduced the morphologic and biochemical estimates of bleomycin-induced lung fibrosis. The hamsters were given pretreatment with deferoxamine and then given bleomycin intratracheally followed by daily doses of the chelator for another 21 days. They had a control group that was given bleomycin alone. The results showed a 33% reduction in lung collagen accumulation in the deferoxamine group vs. the control group; however, some fibrosis was still present in the deferoxamine-bleomycin group [7]. These data suggest that treatment with deferoxamine may reduce the severity of bleomycin lung injury but they do not suggest prevention.

An additional study, set out to see if iron chelation prevents the oxidative stress-mediated toxicity of bleomycin, showed that chelation was not the sole determinant of a compound protecting against bleomycin toxicity [8]. In cases where the fibrosis has already occurred such as in our patient, it may be that the chelation is too late to reverse any damage or change the overall prognosis, but it may be of value in relieving symptoms. Our patient’s breathing and level of comfort improved after receiving two doses of deferoxamine. There is no current data to suggest what dose to use and how many doses to give but this represents an area where further research is needed. It also poses the question of giving deferoxamine before bleomycin therapy and if this would prevent the toxicity and/or affect its ability to act as a chemotherapeutic agent.

## Conclusions

Bleomycin-induced lung injury is a severe and often fatal adverse effect of this chemotherapeutic agent. Iron binds to a structural component of bleomycin and the compound it creates releases free radicals which promote cell death. Currently, there is insufficient clinical evidence to promote the use of iron chelators in bleomycin-induced lung injury. However, they may play a role in preventing pulmonary toxicity and the progression to fibrosis, lessening the physiological severity of the toxicity, or improving symptoms of the toxicity. An additional thought to consider is whether iron infusions to treat iron deficiency anemia in patients also treated with bleomycin should be held within a certain window from the last dose of bleomycin. Further research is desperately needed to answer these questions especially when one considers the poor prognosis of pulmonary fibrosis in these patients.
